# Construction of Efficient Platform *Escherichia coli* Strains for Polyhydroxyalkanoate Production by Engineering Branched Pathway

**DOI:** 10.3390/polym11030509

**Published:** 2019-03-18

**Authors:** Hye-Rim Jung, Su-Yeon Yang, Yu-Mi Moon, Tae-Rim Choi, Hun-Suk Song, Shashi Kant Bhatia, Ranjit Gurav, Eun-Jung Kim, Byung-Gee Kim, Yung-Hun Yang

**Affiliations:** 1 Department of Biological Engineering, College of Engineering, Konkuk University, 120, Neungdong-ro, Gwangjin-gu, Seoul 05029, Korea; hr951205@naver.com (H.-R.J.); tndus4679@naver.com (S.-Y.Y.); ansdbal13@gmail.com (Y.-M.M.); srim1004@gmail.com (T.-R.C.); shs9736@naver.com (H.-S.S.); shashibiotechhpu@gmail.com (S.K.B.); rnjtgurav@gmail.com (R.G.); 2 Institute for Ubiquitous Information Technology and Applications (CBRU), Konkuk University, Seoul 05029, Korea; 3 Institute of Molecular Biology and Genetics, Seoul National University, Seoul 08826, Korea; kej4540@hotmail.com (E.-J.K.); byungkim@snu.ac.kr (B.-G.K.); 4 School of Chemical and Biological Engineering, Seoul National University, 1, Gwanak-ro, Gwanak-gu, Seoul 08826, Korea

**Keywords:** polyhydroxyalkanoate, metabolic engineering, CRISPR/Cas9, *Escherichia coli*, carbon flux distribution, NADPH

## Abstract

Polyhydroxyalkanoate (PHA) is a potential substitute for petroleum-based plastics and can be produced by many microorganisms, including recombinant *Escherichia coli*. For efficient conversion of substrates and maximum PHA production, we performed multiple engineering of branched pathways in *E. coli*. We deleted four genes (*pflb*, *ldhA*, *adhE*, and *fnr*), which contributed to the formation of byproducts, using the CRISPR/Cas9 system and overexpressed *pntAB*, which catalyzes the interconversion of NADH and NADPH. The constructed strain, HR002, showed accumulation of acetyl-CoA and decreased levels of byproducts, resulting in dramatic increases in cell growth and PHA content. Thus, we demonstrated the effects of multiple engineering for redirecting carbon flux into PHA production without any concerns regarding simultaneous deletion.

## 1. Introduction

**Figure 1:**
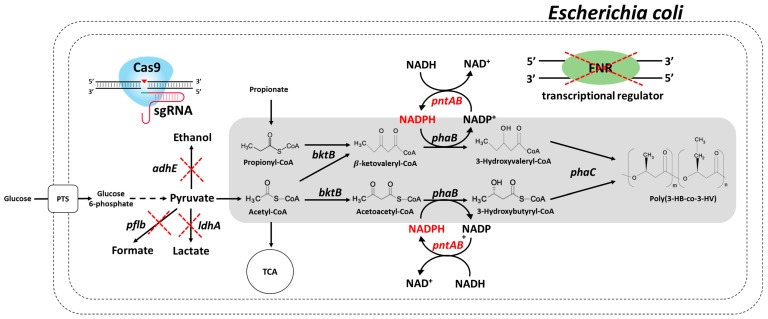
Polyhydroxyalkanoate production in engineered *E. coli*. Engineered metabolic pathways for production of polyhydroxyalkanoate in recombinant *E. coli*. Genes involved in metabolic engineering and polyhydroxyalkanoate production pathway are shown. Pflb, pyruvate formate lyase; LdhA, d-lactate dehydrogenase; AdhE, acetaldehyde-alcohol dehydrogenase; Fnr, transcriptional regulator for expressing anaerobic metabolism; PntAB, transhydrogenase; BktB, β-ketothiolase; PhaB, acetoacetyl-CoA reductase; PhaC, polyhydroxyalkanoate synthase.

Polyhydroxyalkanoate (PHA), a bio-based, biodegradable polymer, is a promising substitute for petroleum-derived plastics [1,2]. In addition, this polymer is biocompatible and, therefore, has several medical applications [3]. Poly(3-hydroxybutyrate) (PHB), the most extensively studied member of the PHA family, is readily produced in several bacteria, including *Ralstonia eutropha*, *Alcaligenes latus*, *Azotobacter vinelandii*, and *Pseudomonas*, as part of their natural metabolism [4,5,6]. Moreover, *Escherichia coli* is an ideal host for PHB production owing to the simplicity of extraction, the absence of an intracellular depolymerization, and the ability to utilize several inexpensive carbon sources [4,7,8]. Recombinant *E. coli* harboring the PHA synthetic operon of *R. eutropha* is shown in Figure 1. After the condensation of two acetyl-CoA molecules to one acetoacetyl-CoA by β-ketothiolase (BktB), which is subsequently reduced to 3-hydroxybutyryl-CoA by NADPH-dependent acetoacetyl-CoA reductase (PhaB), the latter molecule is then polymerized as a monomer to PHB by PHA polymerase (PhaC) [4,9].

PHA production in recombinant *E. coli* is not only a matter of pathway construction but is also affected by many other factors, such as acetyl-CoA and NADPH. Several strategies have focused on enhancing the supply of NADPH and/or acetyl-CoA to improve PHA production [10]. First, acetyl-CoA is a crucial intermediate for PHA production and can directly increase 3-hydroxybuturyl-CoA levels and cell growth. By inactivation of the phosphate acetyltransferase gene (*pta*), which converts acetyl-CoA to acetate, *pta*-deficient *E. coli* induces greater PHA production when compared with wild-type *E. coli* [11]. Another important factor related to PHA production is NADPH, which can be generated through the pentose phosphate (PP) pathway [8]. Overexpression of the glucose-6-phosphate dehydrogenase gene (*zwf*) and 6-phosphodehydrogenase gene (*gnd*) shows enhanced PHA production when compared with the wild-type strain by improving the availability of NADPH [12]. Another strategy to increase PHA productivity in *E. coli* is knockout of the phosphoglucoisomerase gene (*pgi*) [13]. However, there is no report on the effect of multiple engineering of these genes.

The application of clustered regularly interspaced short palindromic repeats (CRISPR) and CRISPR-associated [14] proteins has led to major advancements in molecular microbiology by providing an exciting new genome engineering tool [15,16,17]. Currently, most genetic engineering approaches involve coupling of the CRISPR/Cas9 system with conventional λ-Red recombination as a simplified strategy for chromosomal gene replacement [18]. Because CRISPR/Cas9-coupled recombination overcomes the dependence on the use of chromosome-encoded antibiotic resistance or flippase (Flp) recombination, it allows simpler, less labor-intensive, and efficient production of mutants that are chromosomal “scar”-less [18,19].

In this study, we used CRISPR/Cas9 system to engineer an *E. coli* mutant with deletions of four genes (*pflb*, *ldhA*, *adhE*, and *fnr*) to increase carbon flux into the PHA synthetic pathway. We also overexpressed *pntAB*, which mediates interconversion of NADH and NADPH, for more effective supply of NADPH. We then investigated the effects of multiple engineering for redirecting carbon flux into PHA production. Our results provide important insights into PHA productivity by engineered *E. coli*, resulting in increased intracellular acetyl-CoA, and decreased metabolic byproducts such as lactate and formate.

## 2. Materials and Methods

### 2.1. Bacterial Strains, Media, Reagents, and Culture Conditions

The strains and plasmids used in this study are listed in Table 1. *E. coli* DH5α and BW25113(DE3) were used as host strains for gene cloning and PHB production, respectively. For cell preparation and selection of transformants, the strains were cultured in Luria–Bertani [20] agar and/or liquid broth. LB agar was prepared by dissolving 10 g tryptone, 5 g yeast extract, 10 g NaCl, and 15 g agar in 1 L distilled water. For evaluation of PHB production, transformants were cultured in M9 minimal medium containing 2% glucose, 1 mM MgSO_4_·7H_2_O, and 1 mL/L trace element solution. Trace element solution was prepared with the following composition: 10.0 g/L FeSO_4_·7H_2_O, 2.25 g/L ZnSO_4_·7H_2_O, 0.640 g/L CuSO_4_, 0.560 g/L MnSO_4_·H_2_O, 0.404 g/L (NH_4_)_6_Mo_7_O_24_·4H_2_O, and 10 mL/L of 35% hydrochloric acid. For comparison of poly(3-hydroxybutyrate-*co*-3-hydroxyvalerate) (PHBV) production, cells were also supplemented with 0.15% sodium propionate. Appropriate antibiotics (100 μg/mL spectinomycin and 25 μg/mL chloramphenicol for *E. coli* transformants) were added when required, and 0.1 mM isopropyl-β-d-thiogalactoside (IPTG) was added at the beginning of culture. For preculture, a single colony from an LB agar plate was used to inoculate 3 mL LB medium. The culture was incubated overnight in a shaking incubator at 37 °C and 200 rpm. For flask culture, grown cells were inoculated into 100 mL production medium in 250-mL screwed cap flasks at a dilution of 1:100 (*v*/*v*). The culture was continuously shaken in an incubator at 200 rpm and 30 °C. Restriction enzymes and DNA polymerase were purchased from Enzynomics (Daejeon, Korea), plasmid extraction and gel purification kits were purchased from GeneAll (Seoul, Korea), and medium components were purchased from Bacto or Difco (Sparks, NJ, USA).

### 2.2. Genome Editing

A two-plasmid system, involving pCas9 and pgRNA to direct the modification to the targeted region, was used for genome editing according to a previously reported method [19], with slight modifications. The pCas9 consisted of Cas9 and λ-Red. The pgRNA consisted of the sgRNA sequence and the N_20_ sequence. The pgRNA series, used for single-gene deletion by targeting the N_20_ sequence of the gene, was constructed by inverse polymerase chain reaction (PCR) with the modified N_20_ sequence hanging at the 5′ ends of primers, followed by self-ligation. The editing templates had a 1-kb sequence homologous to each side (upstream and downstream) of the targeted gene in the genome, which was obtained through overlap PCR.

BW25113 (DE3) competent cells harboring pCas were grown in LB medium at 30 °C and induced with 10 mM arabinose for λ-Red induction [25]. For electroporation, 50 μL of cells was mixed with 200 ng of pgRNA series and 100 ng of the editing templates, and then electroporated in a 2-mm Gene Pulser cuvette (Bio-Rad, Hercules, CA, USA) at 2.5 kV. Next, 1 mL LB medium was added. After incubation at 30 °C for 1 h, cells were spread onto LB agar containing kanamycin (50 μg/mL) and ampicillin (100 μg/mL) and then incubated at 30 °C overnight. Transformants were confirmed by colony PCR and DNA sequencing. For curing of the pCas9 and pgRNA series, we used previously reported methods [19].

### 2.3. Analytical Methods

PHB production and quantity were determined using gas chromatography [26] and other methods as previously described [27], with slight modifications. For analysis, culture samples were centrifuged at 10,000× *g* for 10 min, washed with deionized water twice, and suspended in 1 mL water. The suspended samples were subjected to lyophilization, weighed, and placed in Teflon-stoppered glass vials. For methanolysis of PHB samples, 1 mL chloroform and 1 mL of a methanol/sulfuric acid (85:15 *v*/*v*) mixture were added to the vials and then incubated at 100 °C for 2 h. Samples were cooled to room temperature and incubated on ice for approximately 10 min. After adding 1 mL ice-cold water, samples were thoroughly mixed by vortexing for 1 min and centrifuged at 2000× *g*. The organic phase (bottom) was extracted using a pipette and moved to clean borosilicate glass tubes containing anhydrous sodium sulfate (Na_2_SO_4_). Samples were then injected into a GC system (Young Lin Tech, Anyang, Korea) using a DB-Wax column (30 m × 0.32 mm × 0.5 μm; Agilent Technologies, Santa Clara, CA, USA) [28,29]. The split ratio was 1:10. Helium was used as the carrier gas, with a flow rate of 3.0 mL/min. A 2-μL portion of the organic phase was injected using an autosampler. The inlet was maintained at 210 °C. The column oven was held at 80 °C for 5 min, heated to 220 °C at 20 °C/min, and then held at 220 °C for 5 min. Peak detection was performed using a flame ionization detector, which was maintained at 230 °C. The concentrations of furfural were determined using GC, as described above, except for the column oven. The column oven was held at 50 °C for 5 min, heated to 230 °C at 20 °C/min, and then held at 230 °C for 5 min. Glucose consumption and secreted organic acids in culture supernatants were analyzed by high-performance liquid chromatography (Flexar LC, PerkinElmer, Waltham, MA, USA) with a refractive index detector and UV detector at a wavelength of 210 nm, respectively. An Aminex HPX-87H column (Bio-Rad) was used, and the mobile phase in which the flow rate was constant at 0.6 mL/min was 0.008 N H_2_SO_4_. The oven temperature was held constant at 60 °C during operation [30].

### 2.4. CoA Analysis Using Liquid Chromatography (LC)–Mass Spectrometry (MS)

CoA metabolites were extracted using a previously reported method [22,31,32], with slight modifications. Strains YH090 and HR002 were cultured in 50 mL M9 medium containing 2% glucose as a sole carbon source for 6 h at 30 °C. Cells were centrifuged at 10,000× *g* and 4 °C for 10 min and washed with deionized water twice. The supernatants were then discarded. Cells were suspended in 3 mL distilled water and 3 mL ice-cold 20% trichloroacetic acid solution, incubated for 5 min on ice, and again centrifuged at 10,000× *g* and 4 °C for 10 min. Supernatants were filtered using 0.2-μm polyvinylidene difluoride membranes and loaded through an OASIS HLB SPE cartridge for binding and separating CoA thioesters under a vacuum as follows. First, the cartridge was conditioned with ice-cold methanol, followed by 0.1% formic acid in distilled water. The bound CoA metabolites were eluted with methanol and analyzed by UPLC/TQD-MS. Liquid chromatography was performed with the Acquity Ultra Performance LC-system (Waters, Milford, MA, USA) and the Acquity UPLC BEH C18 column (1.7 μm, 2.1 × 100 mm). The temperatures of the column oven and auto-sampler were maintained at 35 and 4 °C, respectively. The mobile phases were (A) 0.1% (*v*/*v*) formic acid in distilled water and (B) 0.1% (*v*/*v*) formic acid in methanol. The linear gradient program began with 80% (A) for 2 min and proceeded to 10% (A) over 2 min, and then returned to initial concentration conditions, which were maintained for 4 min. The total analysis time was 12 min with a flow rate of 0.3 mL/min.

Analytes were detected by multiple reaction monitoring (MRM) using electrospray ionization mass spectrometry (ESI-MS, Waters, Milford, MA, USA). During the run, the system was in positive ESI-mode. The conditions used for the ESI source were as follows: capillary voltage, 3.5 kV; source temperature, 150 °C; and desolvation temperature, 350 °C. Nitrogen was used as the sheath gas with a flow rate of 600 L/h.

## 3. Results

### 3.1. Construction of Engineered E. coli for Redirecting Metabolic Flux into PHA Production

Balancing of metabolic pathway genes is crucial for the efficient conversion of substrates and maximum production of the desired products [33,34]. To avoid byproduct formation, engineering by deleting genes involved in endogenous pathways that contribute to byproduct formation and that share essential intermediates and compete with heterologous production pathways is typically performed [21]. Moreover, accumulation of byproducts such as ethanol, acetate, and other short-chain-length fatty acids hinders the synthesis of nucleic acids, proteins, and lipids, resulting in impairment of cell growth and product formation [35,36].

First, we investigated the effects of multiple engineering for redirecting carbon flux into PHA production and improving NADPH availability. We employed CRISPR-mediated metabolic engineering coupled with the λ-Red recombinase method to rapidly manipulate multiple genes [18,37,38]. Based on our previous report regarding growth and PHA content, we selected BW25113 (DE3) as the *E. coli* host strain for further engineering [39].

Genes involved in metabolic engineering and PHA production are shown in the scheme in Figure 1. To redirect the carbon distribution to PHA production, four genes (*pflb*, *ldhA*, *adhE*, and *fnr*), which contributes to the formation of byproducts such as formate, acetate, lactate, and ethanol, were deleted in *E. coli* BW25113 (DE3) using the CRISPR/Cas9 system. This deleted strain was named HR100. To further improve NADPH availability for PHA production, *pntAB*, which catalyzes the interconversion of NADH and NADPH, was overexpressed in HR100; the resulting strain was named HR200. As *E. coli* host strains for PHA production, HR100 and HR200 were used after introduction of pLW487 (harboring *bktB*, *phaB*, and *phaC* from *R. eutropha*) [28,29]. The resulting strains were named HR001 and HR002, respectively. We used both these strains (HR001 and HR002) in further experiments for comparison of PHA production and metabolite analysis.

### 3.2. PHB Production and Metabolite Analysis in Engineered E. coli

**Figure 2:**
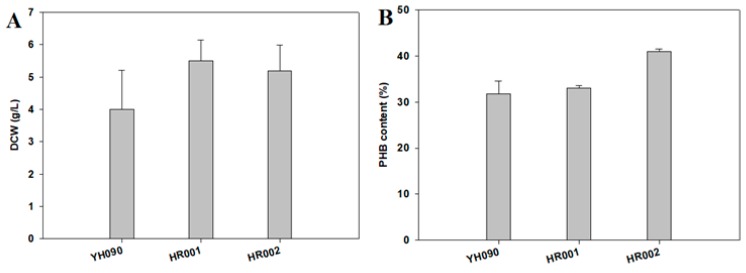

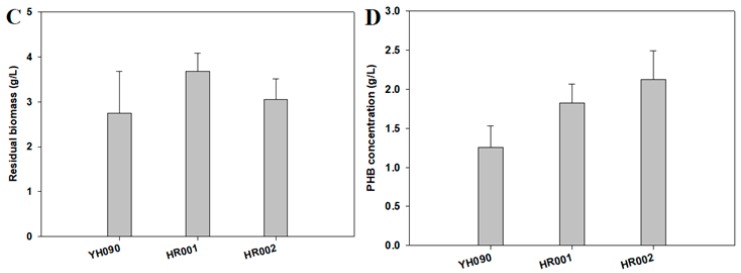
Polyhydroxybutyrate (PHB) production in engineered *E. coli*: (**A**) DCW (dry cell weight, g/L); (**B**) PHB content (wt/wt %); (**C**) residual biomass (g/L); and (**D**) PHB concentration (g/L). Cell cultivation was conducted for 48 h in M9 minimal medium containing 2% glucose as a sole carbon source. Error bars represent the standard deviations of two replicates.

To confirm the effects of multiple deletions and overexpression, we monitored cell growth and PHB production in strains HR001 and HR002 (Figure 2). Cell cultivation was conducted for 48 h in M9 minimal medium containing 2% glucose as a sole carbon source. Both constructed strains (HR001 and HR002) showed dramatic increases in cell growth and PHB content compared with those of strain YH090, without any concern regarding modification of chromosomal DNA sequences. Compared with HR001, HR002 showed slightly decreased cell growth from 5.5 to 5.2 g/L as dry cell weight (DCW; Figure 2A). However, PHB content was increased from 32% to 41%, and PHB concentration was increased from 1.8 to 2.1 g/L (Figure 2D). Because *pntAB* was also overexpressed in addition to deletion of the four genes, we concluded that increased NADPH supply led to accelerated PHB production in HR002 compared with YH090 and HR001.

**Figure 3:**
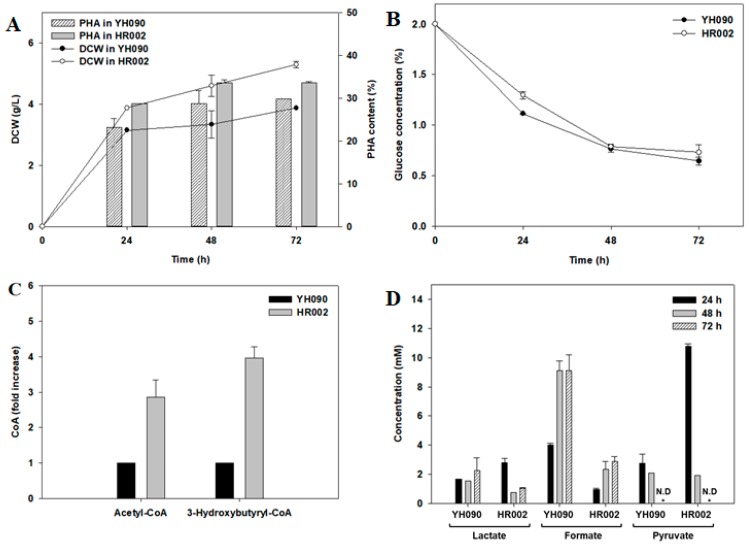
Polyhydroxybutyrate (PHB) production in engineered *E. coli* over time: (**A**) DCW (dry cell weight, g/L) and PHB content (wt/wt %); (**B**) glucose concentration (g/L); (**C**) relative amount of CoA metabolites (fold increase); and (**D**) secreted organic acid concentration [40]. Cell cultivation was conducted for 72 h in M9 minimal medium containing 2% glucose as a sole carbon source. CoA metabolites were measured at 6 h. Error bars represent the standard deviations of two replicates.

For further investigation, CoA metabolites (acetyl-CoA and 3-hydroxybutyryl-CoA), secreted organic acids (lactate, formate, and pyruvate), and glucose consumption were monitored in addition to cell growth and PHB production in strains YH090 and HR002 (Figure 3). Cells were cultivated in M9 minimal medium containing 2% glucose as a sole carbon source for 72 h. CoA metabolites were evaluated at 6, 12, and 24 h. However, meaningful amounts of CoA metabolites were not detected at 12 or 24 h. The fold increase in CoA metabolites was calculated as CoA (fold) = $\frac{CoA_{HR002}}{CoA_{YH090}}\times 100$, where CoA is the CoA metabolite concentration.

As described above, HR002 showed higher cell growth and PHB production than YH090 (Figure 2, Figure 3A). Interestingly, glucose consumption was similar in strains YH090 and HR002 for 72 h (Figure 3B). Acetyl-CoA and 3-hydroxybutyryl-CoA are key building blocks and precursors in PHB production [22]. CoA metabolites involved in PHB production were significantly increased in HR002, with a 2.8-fold increase in acetyl-CoA and a 4-fold increase in 3-hydroxybutyryl-CoA at 6 h (Figure 3C). Lactate and formate are major byproducts that are mostly secreted into the culture broth [41]. During the initial 24 h of cultivation, lactate in HR002 was slightly higher than that in YH090. However, after 24 h of the initial cultivation, lactate was decreased in HR002, and the final concentrations detected were 1.05 mM lactate in strain HR002 and 2.27 mM lactate in strain YH090 (Figure 3D). Formate was maintained at a lower level in strain HR002 during the 72 h cultivation, and the final concentrations detected were 2.89 mM formate in strain HR002 and 9.12 mM formate in strain YH090. Pyruvate in strain HR002 was significantly increased by 3.9-fold to 10.77 mM during the initial 24 h of cultivation, however, after 48 h of the initial cultivation, pyruvate was not detected in both strains. In terms of accumulated CoA metabolites and decreased byproducts formation, carbon flux may be redirected to PHB production rather than byproducts formation. In other words, engineering of the branched pathway resulted in decreased formation of byproducts, increased CoA metabolites, and increased PHB production. Accordingly, we demonstrated the effects of multiple engineering for redirecting carbon flux to PHB production without any concerns regarding simultaneous deletion.

### 3.3. Application of the Engineered E. coli for PHBV Production

Poly(3-hydroxybutyrate) (PHB) is the most extensively studied member of the PHA family [6]. The later discovered poly(3-hydroxybutyrate-*co*-3-hydroxyvalerate) (PHBV) has been considered as an attractive co-polymer that owns better biocompatibility and mechanical properties [39,40]. For the production of PHBV, the 3HV (3-hydroxyvalerate) precursor, such as propionate or valerate, is typically added to the culture medium [30,39]. However, supplementation with related precursors inhibits cell growth [21,30]. In addition to secondary carbon sources, there have been many reports describing metabolic engineering for a high propionyl-CoA/acetyl-CoA ratio or a high concentration of 3-hydroxyvaleryl-CoA [21,26,30,39,41].

**Figure 4:**
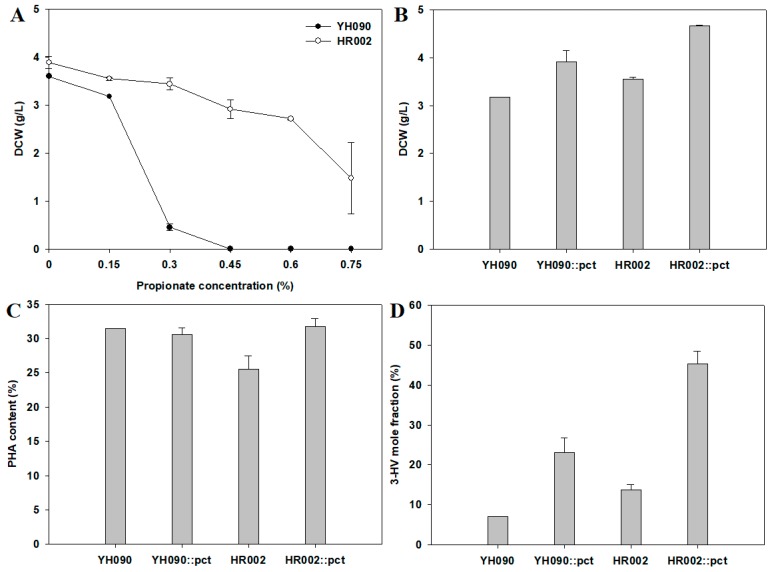
Propionate resistance and the effects of CoA transferase on the high 3-hydroxyvalerate mole fraction of PHBV: (**A**) DCW (dry cell weight, g/L) according to the concentration of propionate; (**B**) DCW (dry cell weight, g/L); (**C**) PHA content (wt/wt %); and (**D**) 3-hydroxyvalerate monomer content (mol %). Cell cultivation was conducted for 72 h in M9 minimal medium containing 2% glucose and different concentrations of propionate (0–0.75% in (**A**); and 0.15% in (**B**–**D**)). Error bars represent the standard deviations of two replicates.

As described above, strain HR002 showed increased cell growth and redirected carbon flux to PHB production by multiple engineering of the branched pathway. To examine whether strain HR002 may have applications in PHBV production, we performed a comparison of cell growth in the presence of different concentrations of propionate (0–0.75%) in strains YH090 and HR002 (Figure 4A). In contrast to significantly decreased cell growth in strain YH090, strain HR002 showed robust cell growth, suggesting that engineering of the branched pathway could contribute to resistance to high concentrations of supplemented organic acids owing to the reduced excretion of organic acids (Figure 3D). However, the 3-HV mole fraction of PHBV produced from strain HR002 was still lower than 20% (Figure 4D).

Based on this result, we introduced propionyl-CoA transferase (Pct) from *R. eutropha* H16 to strain HR002 and added propionate as the precursor substrate for the production of PHBV based on previous reports [21,40]. Cell cultivation was conducted for 72 h in M9 minimal medium containing 2% glucose and 0.15% sodium propionate.

Although there was no notable difference in PHA content, the 3-HV mole fraction was significantly improved in both YH090::*pct* and HR002::*pct* compared with that in strains lacking Pct (Figure 4C,D). These results were consistent with previous reports demonstrating that Pct is directly involved in propionyl-CoA synthesis and further incorporation of 3-HV in PHA [21,41]. Additionally, HR002::*pct* showed a 2.0-fold increase in the 3-HV mole fraction compared with YH090::*pct*, yielding a final 3-HV mole fraction of 45%. As a result, strain HR002 could be applied for the production of PHBV with introduction of *pct*.

**Figure 5:**
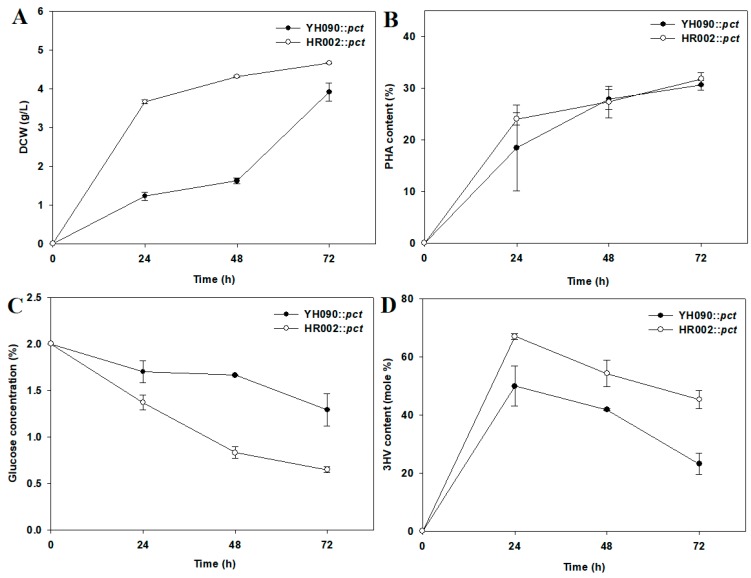
Monitoring of PHBV production in YH090::*pct* and HR002::*pct*: (**A**) DCW (dry cell weight, g/L); (**B**) PHA content (wt/wt %); (**C**) Glucose concentration (g/L); and (**D**) 3-hydroxyvalerate monomer content (mol %). Cell cultivation was conducted for 72 h in M9 minimal medium containing 2% glucose and 0.15% sodium propionate. Error bars represent the standard deviations of two replicates.

To examine further availability for PHBV production, cell growth, PHA content, glucose consumption, and 3-HV mole fraction were monitored in YH090::*pct* and HR002::*pct* for 72 h (Figure 5). Cells were cultivated in M9 minimal medium containing 2% glucose and 0.15% propionate. HR002::*pct* showed faster cell growth than YH090::*pct* for 72 h, with a reduced lag phase (Figure 5A). As described above, decreased byproduct formation in strain HR002 may influence resistance to excess organic acids owing to increased PHBV production (Figure 3 and Figure 5). Interestingly, HR002::*pct* showed significantly accelerated glucose consumption compared with YH090::*pct* (Figure 5C). This may result in sufficient production of acetyl-CoA and propionyl-CoA, leading to increased production of PHBV containing a higher 3-HV mole fraction (Figure 5D).

Further, we compared the productivity (3-HV yield from propionate, Y_3HV/Prop_) of HR002::*pct* with other reported PHBV producing strains (Table 2). A significantly increased 3-HV from propionate was achieved by HR002::*pct*, reaching Y_3HV/Prop_ = 0.67 g/g, while YH090::*pct* showed Y_3HV/Prop_ = 0.34 g/g in this study. Consequently, strain HR002::*pct* may have applications in the production of PHBV and PHB by redirecting carbon flux into PHA production. In addition, this multiple engineering approach could have applications in microbial production of other chemicals, such as alcohols and terpenoids.

## 4. Discussion

The increasing preference for bioplastics worldwide is an important driver for many fields of research. To date, PHA has been shown to be produced by many microorganisms, including *R. eutropha*, *A. latus*, and recombinant *E. coli* using diverse inexpensive carbon sources [4,7,8]. In addition to pathway construction, many studies also focus on enhancing the supply of acetyl-CoA and/or NADPH [10].

In this study, we reported the effects of multiple engineering for balancing metabolic pathways to avoid byproduct formation. We deleted four genes (*pflb*, *ldhA*, *adhE*, and *fnr*), which contribute to the formation of byproducts, using the CRISPR/Cas9 system. In addition to the simultaneous deletion, we overexpressed *pntAB*, which catalyzes the interconversion of NADH and NADPH. The constructed strain, HR002, showed dramatic increases in cell growth and PHB contents compared with the control strain. In addition, strain HR002 exhibited accumulation of CoA metabolites and decreased formation of byproducts. Accordingly, engineering of the branched pathway resulted in carbon flux distribution to PHA production and reinforced NADPH supply without any concerns regarding simultaneous deletion. This multiple engineering approach could also be applied for microbial production of other types of chemicals.

## Figures and Tables

**Table 1:** Bacterial strains, plasmids, and primers used in this study.

Strain or Plasmid	Description	Reference
*E. coli* strains		
DH5α	General cloning strain	Invitrogen
BW25113	*lacI*^q^, *rrnB3*, ∆*lacZ4787*, *bsdR514*, ∆*araBAD*, ∆*rhaBAD*	[21]
BW25113(DE3)	BW25113 derivative containing DE3	[22]
YH090	BW25113 (DE3) containing pLW487	[22]
HR100	BW25113 (DE3), ∆*pflb*, ∆*ldhA*, ∆*adhE*, ∆*fnr*	This study
HR200	BW25113 (DE3), ∆*pflb*, ∆*ldhA*, ∆*adhE*, ∆*fnr*, containing pACYC::*pntAB*	This study
HR001	BW25113 (DE3), ∆*pflb*, ∆*ldhA*, ∆*adhE*, ∆*fnr*, containing pLW487	This study
HR002	HR001 containing pACYC::*pntAB*	This study
Plasmids		
pLW487	Spectinomycin-resistant pEP2-based plasmid carrying PCR products of *bktB*, *phaB*, and *phaC* under the *trc* promoter from *Ralstonia eutropha* H16	[23]
pACYCDuet-1	A compatible chloramphenicol-resistant plasmid carrying the T7 promoter	Novagen
pACYC::*pntAB*	pACYCDuet-1 carrying the PCR product of *pntAB* from *E. coli* K12 MG1655	[24]
pET-28a	A compatible kanamycin-resistant plasmid carrying the T7 promoter	Novagen
pET-28a::*pct*	pET-28a carrying the PCR product of *pct* from *Ralstonia eutropha* H16	This study
pCas	*repA101* (Ts), kanamycin-resistant, *cas9* gene, *Red* recombination genes under an arabinose-inducible promoter, *lacI*^q^, P_trc_-sgRNA targeting pBR322 origin of pgRNA	This study
pgRNA	pBR322 origin, ampicillin-resistant, sgRNA with an N_20_ sequence for targeting gene	This study
Primers		
N_20__*pflb*_F	CTCT ACTAGT CATCGTATTCCGGAGTACGC GTTTTAGAGCTAGAAATAGC	This study
N_20__*ldhA*_F	CTCT ACTAGT TTAAACCAGTTCGTTCGGGC GTTTTAGAGCTAGAAATAGC	This study
N_20__*adhE*_F	CTCT ACTAGT CCGAAAGCACACAGGGACTT GTTTTAGAGCTAGAAATAGC	This study
N_20__*fnr*_F	CTCT ACTAGT TTCCGCCTGACGATGACTCG GTTTTAGAGCTAGAAATAGC	This study
N_20__R	CTCT ACTAGT ATTATACCTAGGAC	This study

**Table 2:** Comparison of the reported PHBV producing *E. coli*.

*E. coli* Strain	Carbon Source	DCW (g/L)	PHA Content (wt %)	3-HV (mol %)	Y_3HV/Prop_ (g 3-HV/g Propionate)	Cultivation	Reference
YH090::*pct* (BW25113 (DE3) containing pLW487 (PHA operon) and *pct* from *C. propionicum*)	1% glucose, 0.3% propionate	2	50	80	0.30	Batch (Flask)	[39]
YH090::*sucCD* (BW25113 (DE3) containing pLW487 (PHA operon) and *sucCD*)	1% glucose, 0.3% propionate	1.2	54.8	24.1	0.09	Batch (Flask)	[28]
YH090::*pct* (BW25113 (DE3) containing pLW487 (PHA operon) and *pct*from *R. eutropha* H16)	2% glucose, 0.15% propionate	3.9	30.6	23.1	0.34	Batch (Flask)	This study
HR002::*pct* (BW25113 (DE3) containing pLW487 (PHA operon), *pntAB*, *pct* from *R. eutropha* H16, ∆*pflb*, ∆*ldhA*, ∆*adhE*, ∆*fnr*)	2% glucose, 0.15% propionate	4.5	32	50	0.67	Batch (Flask)	This study
LS5218 containing p4A (PHA operon), *fadR*, *atoC*	0.4% glucose, 25 mM propionate	-	-	39	0.10	Batch (Flask)	[42]
BL21 containing pLW487 (PHA operon) and *atoAD*	1% glucose, 0.3% propionate	3	35	70	0.30	Fed-batch (Flask)	[32]
XL10 containing pBHR68 (PHA operon) and *prpP*	2% glucose, 0.2% propionate	39.8	60.5	15.1	0.46	Fed-batch (Fermentor)	[38]
